# Evaluating the effectiveness of traumatic brain injury state laws among high school athletes

**DOI:** 10.1186/s40621-020-00241-6

**Published:** 2020-04-13

**Authors:** Alan T. Arakkal, Anna E. Barón, Molly M. Lamb, Sarah K. Fields, R. Dawn Comstock

**Affiliations:** 1 Department of Epidemiology, College of Public Health, University of Iowa, 145 N Riverside Dr 100 CPHB, Iowa City, IA 52242 USA; 2 Department of Biostatistics and Informatics, Colorado School of Public Health, University of Colorado Denver-Anschutz Medical Campus, Aurora, Colorado USA; 3 Department of Epidemiology, Colorado School of Public Health, University of Colorado Denver-Anschutz Medical Campus, Aurora, Colorado USA; 4 Department of Communication, University of Colorado Denver, Denver, Colorado USA

**Keywords:** Injury, Concussion, Traumatic brain injury, Traumatic brain injury laws, Profile likelihood approach, Public health

## Abstract

**Background:**

Traumatic brain injury legislation varies across states. A comprehensive nationwide evaluation of state traumatic brain injury laws is vital given growing populations of high school athletes. This study evaluates the effectiveness of traumatic brain injury laws by examining longitudinal trends in incident and recurrent concussion rates and determines if state level variations in legislation’s language affected the observed trends.

**Methods:**

In this retrospective ecological study of a large national sample of US high schools from 2005/06 through 2017/18, piecewise regression models along with a profile likelihood approach were utilized to examine longitudinal trends in incident and recurrent concussion rates.

**Results:**

Overall incident concussion rates increased by an additional 1.85%/standardized month (STDM) (95% confidence interval (CI): 1.14, 2.56%) prior to law passage and decreased by an additional 1.08%/ STDM (95%CI: − 1.43, − 0.72%) after law passage. Similar trends were observed for overall recurrent concussion rates. Among states that specified the category of healthcare provider for return to play clearance, post-law recurrent concussion rates decreased on average by an additional 1.59%/STDM (95%CI: − 3.42, 0.22%) compared to states that did not specify the category of healthcare provider.

**Conclusions:**

The passage of state level traumatic brain injury laws was associated with an increase in overall incident and recurrent concussion rates prior to law passage and a decrease in rates after law passage. Although not statistically significant, states with traumatic brain injury laws specifying the category of healthcare provider for return to play clearance had a greater rate of decline in post-law recurrent concussion rates compared to states not specifying the category of healthcare provider. The findings suggest that state traumatic brain injury laws may benefit from specifying the category of healthcare provider allowed to provide return to play clearance, if they do not already include such language.

## Background

In the United States (US), approximately 8 million high school athletes participate in over 60 different school sanctioned sports annually (The National Federation of State High School Associations 2018). Participation in high school sports has increased steadily over time (The National Federation of State High School Associations 2018). As participation increases, sports related injuries, including concussions, may also increase. A study of 25 US high schools over an 11-year period showed concussion rates increased 15.5% annually (Lincoln et al. 2011).

Concussions, a mild form of traumatic brain injury (TBI) (Centers for Disease Control and Prevention 2018), may go undetected because of symptom variability, lack of education regarding concussion signs and symptoms, etc. (McCrea et al. 2004; Fedor and Gunstad 2015; Williamson and Goodman 2006) Undiagnosed concussions may place athletes at risk for subsequent brain injuries if they return to play (RTP) before they are adequately recovered. Symptoms associated with recurrent concussions, including the possibility of second impact syndrome (Cantu 1998), may be more severe and require a longer recovery time than those associated with incident concussions (Collins et al. 2002; Guskiewicz et al. 2003).

State laws were implemented to address the potentially devastating consequences of TBIs occurring in youth sports activities (Harvey 2017). The first TBI law, the Zackery Lystedt Law, passed in 2009 in Washington State contains three major components: (1) removing athletes with a suspected head injury from play; (2) requiring a licensed healthcare provider trained in the evaluation and management of concussions to approve an athlete’s RTP; and (3) requiring education for coaches, parents/guardians, and youth athletes about concussion symptoms (Harvey 2017). By 2014, all 50 states and the District of Columbia had passed TBI laws, but specific law language varied across states. For example, Rhode Island requires a licensed physician to provide an athletes RTP clearance; whereas in Iowa, an athletic trainer (AT) can provide RTP clearance (Harvey 2017).

While state-specific studies examining the effects of TBI laws on concussion rates exist (Mackenzie et al. 2015; O’Kane et al. 2014; LaRoche et al. 2016; Bompadre et al. 2014), only a few have studied the effects of the state laws nationwide (Yang et al. 2017; Gibson et al. 2015). Several state-specific studies found a two-fold increase in concussion rates after the state’s TBI law passage (Mackenzie et al. 2015; Bompadre et al. 2014; Yang et al. 2017; Gibson et al. 2015), while others found no significant changes in concussion rates after passage (O’Kane et al. 2014; LaRoche et al. 2016). A full understanding of the effectiveness of TBI laws has been limited because existing studies have only analyzed concussion trends for a short time period, have not differentiated between incident and recurrent concussions, and/or have not accounted for the variation in the language used in TBI laws across states.

This study’s objective was to evaluate the effectiveness of state level TBI laws by examining longitudinal trends in incident and recurrent concussion rates. A secondary objective was to determine if state level variations in legislation’s language influenced observed trends. We hypothesized that incident and recurrent concussion rates increased in the first year after passage of state level TBI laws, followed by a decrease in recurrent concussion rates and a stabilization in incident concussion rates 2 years after law passage. Additionally, trends in concussion rates were hypothesized to differ when stratifying by the specific TBI legislation language.

## Methods

### Data sources

Data from the 2005/06 through 2017/18 academic years was obtained from the National High School Sports-Related Injury Surveillance Study (High School RIO), a large national prospective internet-based sports injury surveillance system (High School RIO 2018). High schools with National Athletic Trainers’ Association-affiliated certified athletic trainers were eligible to participate (High School RIO 2018; Comstock et al. 2018). The methodology of High School RIO has been previously described (Yang et al. 2017; Kerr et al. 2018).

State-specific TBI law information was obtained from LawAtlas, an online legal mapping tool that collects and tracks public health laws and policies (Harvey 2017). LawAtlas’ youth sport TBI dataset contains state-specific legal text of the law across particular moments in time, including initial law passage dates as well as dates of implementation. Initial law passage date and information was utilized in this study as opposed to law implementation because media coverage of and public notification about new TBI laws was almost always greatest at the time of the law’s passage.

### Selection criteria

All concussions captured by High School RIO from 2005/06 through 2017/18 were evaluated (*n* = 17,336). Concussions missing date of injury (*n* = 467), state (*n* = 9), injury classification (i.e. incident or recurrent) (*n* = 162), or missing athletic exposure (AE) data (*n* = 7) were excluded. Concussions with injury type classified as unknown (*n* = 1), other (*n* = 21), or as a recurrent injury that occurred from a non-sport related mechanism (e.g. car accident, etc.) (*n* = 6) were also excluded. A total of 666 (3.8%) concussions were excluded. Concussion rates were calculated using the state in which the student athlete’s school was located rather than the location where the injury occurred because the latter is not available in the High School RIO database.

### Data management

Data management was performed using SAS, version 9.4 (SAS Institute Inc., Cary, North Carolina). Athletic exposures and concussions were summed across four-week increments from exposure weeks 1 through 52 for each state by academic year. States were categorized into groups using initial law passage information based on state level variations in legislation’s language pertaining to RTP clearance and education requirements. For RTP clearance requirements, states were dichotomized into: (1) those specifying the category of healthcare provider (e.g., physicians, nurses, physician’s assistants, ATs, etc.); or (2) those not specifying the category of healthcare provider eligible to provide RTP clearance and Illinois, the only state at law passage that did not require athletes to receive RTP clearance from healthcare providers. Additionally, states specifying the category of healthcare provider were further dichotomized into: (1) those that allowed multiple categories of healthcare providers (e.g., physicians, nurses, physician’s assistants, ATs, etc.); or (2) those that specified only physicians were eligible to provide RTP clearance. For education requirements, states were dichotomized into: (1) those requiring education regarding concussion signs and symptoms for both coaches and parents/guardians; or (2) those requiring education for coaches or parents/guardians, but not both.

A standardized month (STDM) variable was created to account for the variation in the timing of law passage across states, calculated by the difference in months between a state’s TBI law passage date and the date of a reported concussion. Concussions reported during the month of the relevant state law passage were categorized as 0. One negative and positive STDM unit represents concussions occurring 1 month before and 1 month after law passage, respectively.

### Data analysis

Data analysis was performed using SAS, version 9.4 (SAS Institute Inc., Cary, North Carolina). Fourteen generalized linear mixed-effects piecewise regression models with a negative binomial distribution were employed to test differences in incident and recurrent concussion rates before and after TBI law passage. This includes two models for overall incident and recurrent concussion rates, four incident and recurrent models comparing states specifying vs. not specifying the category of healthcare provider eligible to provide RTP clearance, four incident and recurrent models comparing states that allowed multiple categories of healthcare providers vs. those that specified only physicians were eligible to provide RTP clearance, and four incident and recurrent models comparing states requiring education regarding concussion signs and symptoms for both coaches and parents/guardians vs. those requiring education for coaches or parents/guardians, but not both. A random intercept for state was included to account for the single-level clustering and correlation.

The profile likelihood approach, a robust statistical method used to identify unknown change points in piecewise regression models, was employed in this study (Muggeo 2003; Tishler and Zang 1981; Stasinopoulos and Rigby 1992; Celermajer et al. 1994; Hall et al. 2000). The method is utilized in various fields of research including, but not limited to, infectious disease, chronic disease, and surveillance related studies (Muggeo 2003; Celermajer et al. 1994; Hall et al. 2000). The appeal of the profile likelihood approach is that the method does not require a priori knowledge of the timing or location during which a shift in the piecewise regression model occurs (Muggeo 2003; Tishler and Zang 1981). The method identifies change points based on maximizing the likelihood for the data observed. Methods for applying the profile likelihood approach are described in previous research (Muggeo 2003; Tishler and Zang 1981; Stasinopoulos and Rigby 1992; Hall et al. 2000). In this study, a number of factors could have impacted the location of the change points, such as injury categorization (i.e. incident or recurrent), specific language of the TBI laws, media coverage, etc. Thus, the true location of the change points could not safely be assumed to be known a priori.

Two knots (i.e. change points) were incorporated into each model to allow for trends in concussion rates to change immediately after law passage and during the post-law period. This was accomplished by including the following quantities in each model: (STDM - τ_1_)^+^ and (STDM - τ_2_)^+^, where τ_1_ is the change point immediately after law passage and τ_2_ is the change point during the post-law period. For (STDM - τ_1_)^+^, the knot term was zero for all concussions with a STDM of less than or equal to τ_1_ and the knot term was equal to STDM - τ_1_ for all concussions with a STDM of greater than τ_1_. Similarly, for (STDM - τ_2_)^+^, the knot term was zero for all concussions with a STDM of less than or equal to τ_2_ and the knot term was equal to STDM - τ_2_ for all concussions with a STDM of greater than τ_2_. In other words, two knots were incorporated into the models to allow for the relationship between the outcome variable (Y) and the explanatory variable (X) to have different linear relationships at different ranges of X. The knots can be thought of as values of X at which the linear relationship between Y and X changes.

The profile likelihood as a function of τ_1_ and τ_2_ was calculated by maximizing the multivariate log likelihood with respect to all other parameters in the model. This was done over a range of six-month increment combinations of τ_1_ and τ_2_ selected for evaluation. For example, in this study, reported incident concussions occurred over a range of − 96 through + 108 STDMs, which resulted in the evaluation of 595 unique six-month increment combinations of τ_1_ and τ_2_. The combination of τ_1_ and τ_2_ that resulted in the largest profile likelihood was the maximum likelihood estimate for τ_1_ and τ_2_. These values were selected as the change points for the model and applied as τ_1_ and τ_2_ for the knot quantities in the piecewise regression model. In other words, to identify the optimal placement of the knots, multiple models were fit (in the incident concussion example above 595 models were assessed) by varying the placement of the two knots based on all possible six-month combinations. The log likelihood was obtained for each model assessed. The model that resulted in the largest log likelihood of all models evaluated was selected as the optimal model and the knot values for that specific model were then applied to the final model that was used for inference. The analysis was stratified by injury classification (i.e. incident or recurrent) and TBI legislation language groups. An alpha of 0.05 was selected as the level of significance. The estimated rates of change in concussion rates pre and post change points and their corresponding confidence intervals were reported for all outcomes modeled. Differences in rates of change in concussion rates pre and post change points were reported by injury classification and TBI legislation language groups. Additionally, a two-sample independent t-test was employed to test the differences in estimated rates of change in concussion rates between the types of injury classification, as well as, TBI law language groups.

## Results

The study dataset contained 16,670 concussions: 15,153 (90.9%) incident and 1517 (9.1%) recurrent. A comparison of the distribution of states by specific TBI legislation language is presented in Table 1. The study dataset contained concussion data for 49 states, including the District of Columbia.

**Table 1:** Distribution of States by Specific Language of TBI Laws at Law Passage

**TBI Law Language**	**States**^**a**^	**LawAtlas Dataset** **n (%)**	**High School RIO Dataset** **n (%)**
***Specific Language of TBI Laws Relating to What Category of Healthcare Provider Was Legally Allowed to Provide Clearance for an Injured Athlete to Return to Play***			
Specified the Category of Healthcare Provider for RTP Clearance	AK, **AL, AZ, CO, CT, DE, GA, HI, IA, ID, KS, LA, MA, ME, MN, MS, NC, NE, NJ, NM, NV, NY, OH, RI, SC, TN, TX**	27 (52.9%)	26 (53.1%)
* Physicians Only*	**AL, DE, NY, OH, RI, TN, TX**	7 (25.9%)^c^	7 (26.9%)^c^
* Multiple Categories of Healthcare Providers*^b^	AK, **AZ, CO, CT, GA, HI, IA, ID, KS, LA, MA, ME, MN, MS, NC, NE, NJ, NM, NV, SC**	20 (74.1%)^c^	19 (73.1%)^c^
Did Not Specify the Category of Healthcare Provider for RTP Clearance	**AR, CA, DC, FL, IL, IN, KY, MD, MI, MO, MT, ND, NH, OK, OR, PA, SD, UT, VA, VT, WA, WI, WV,** WY	24 (47.1%)	23 (46.9%)
***Specific Language of TBI Laws Relating to Who Was Required to Complete Education Regarding Concussion Signs and Symptoms***			
Specified Education for Both Coaches and Parents/Guardians	AK**, AL, AR, AZ, DE, FL, HI, IA, ID, IL, IN, KS, KY, LA, MA, MD, ME, MI, MT, NC, ND, NH, NJ, NM, NY, OH, OK, PA, RI, SC, SD, TN, TX, UT, VA, VT, WA, WI, WV**, WY	40 (78.4%)	38 (77.6%)
Specified Education for Either Coaches or Parents/Guardians	**CA, CO, CT, DC, GA, MN, MO, MS, NE, NV, OR**	11 (21.6%)	11 (22.4%)
***State-Specific Year of TBI Law Passage***			
2009	**OR, WA**	2 (3.9%)	2 (4.1%)
2010	**CT, ID, MA, NJ, NM, OK, RI, VA**	8 (15.7%)	8 (16.3%)
2011	AK, **AL, AZ, CA, CO, DC, DE, IA, IL, IN, KS, LA, MD, ME, MN, MO, NC, ND, NE, NV, NY, PA, SD, TX, UT, VT,** WY	27 (52.9%)	25 (51.0%)
2012	**FL, HI, KY, MI, NH, OH, WI**	7 (13.7%)	7 (14.3%)
2013	**AR, GA, MT, SC, TN, WV**	6 (11.8%)	6 (12.2%)
2014	**MS**	1 (2.0%)	1 (2.0%)
**Total**		**51 (100.0%)**	**49**^**d**^**(100.0%)**

*Abbreviations*: *RTP* Return to play, *TBI* Traumatic brain injury

^a^ Includes the District of Colombia. States captured in the High School RIO dataset are bolded

^b^ Includes categories such as physicians, nurses, physician’s assistants, athletic trainers, etc.

^c^ Proportion of total number of states that specified category healthcare provider for return to play clearance

^d^ States not captured in the High School RIO dataset include AK and WY

### Trends in concussion rates

#### Overall

**Fig. 1:**
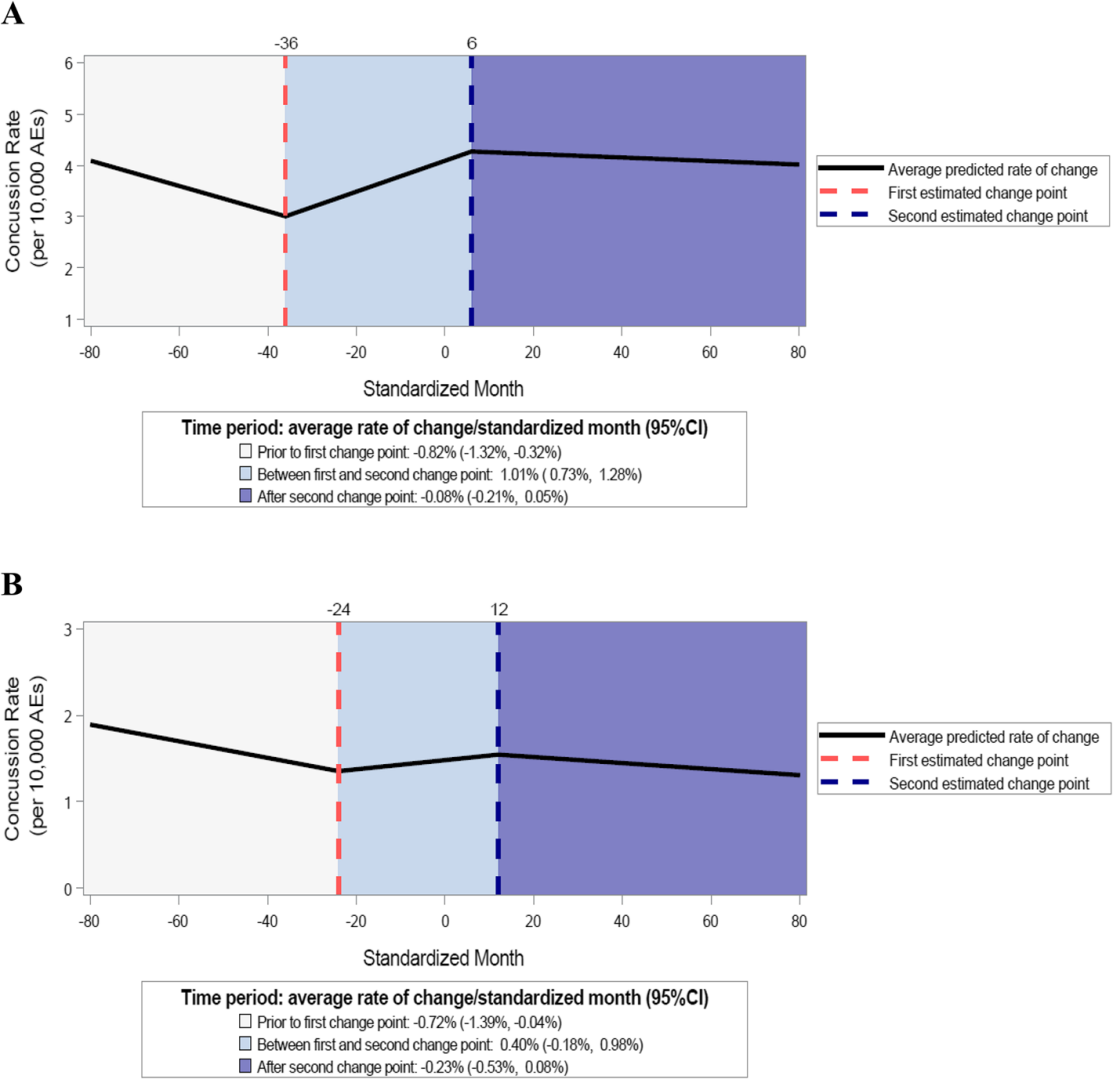
Estimated trends, 2005/06–2017/18. **a** Incident concussion rates; **b** recurrent concussion rates

Overall incident concussion rates increased on average by an additional 1.85%/STDM (95% confidence interval (CI): 1.14, 2.56%) prior to law passage and decreased on average by an additional 1.08%/STDM (95%CI: − 1.43, − 0.72%) after law passage. Overall recurrent concussion rates increased on average by an additional 1.12%/STDM (95%CI: 0.00, 2.26%) prior to law passage and decreased on average by an additional 0.62%/STDM (95%CI: − 1.40, 0.16%) after law passage (Table 2 and Fig. 1a and b). Although not statistically significant, the rate of decline during the post-law period for recurrent concussion rates was 0.15%/STDM (95%CI: − 0.48, 0.19%) greater compared to incident concussion rates (Table 2).

**Table 2:** Estimated Changes in Concussion Rates by Time Period and Specific Language of TBI Laws, 2005/06–2017/18

**Specific Language of TBI Laws**	**After First Change Point**						**After Second Change Point**					
	**Incident**^**a**^	**95%CI**	**Recurrent**^**a**^	**95%CI**	**Incident vs. Recurrent**^**b**^	**95%CI**	**Incident**^**a**^	**95%CI**	**Recurrent**^**a**^	**95%CI**	**Incident vs. Recurrent**^**b**^	**95%CI**
Overall	1.85%*	1.14, 2.56%	1.12%*	0.00, 2.26%	− 0.61%	− 1.24, 0.03%	− 1.08%*	− 1.43, − 0.72%	− 0.62%	− 1.40, 0.16%	− 0.15%	− 0.48, 0.19%
Specified Category of Healthcare Provider for RTP Clearance	2.03%*	0.86, 3.21%	1.86%*	0.68, 3.06%	0.71%	− 0.23, 1.66%	− 0.78%*	− 1.16, − 0.39%	− 3.29%*	− 5.57, − 0.96%	− 1.84%*	− 3.55, − 0.09%
Did Not Specify Category of Healthcare Provider for RTP Clearance	8.28%*	4.93, 11.75%	5.77%*	0.57, 11.24%	− 2.31%	− 7.46, 3.13%	− 7.35%*	− 10.02, − 4.59%	− 5.54%*	− 10.01, − 0.86%	− 0.41%	− 0.84, 0.03%
Specified Multiple Categories of Healthcare Providers for RTP Clearance	2.77%*	0.90, 4.68%	3.21%*	0.47, 6.02%	2.33%	− 0.20, 4.93%	− 0.41%	− 0.84, 0.02%	− 4.51%	− 9.00, 0.21%	− 1.87%	− 4.52, 0.85%
Specified Physicians Only for RTP Clearance	− 0.91%*	− 1.61, − 0.21%	2.51%*	0.06, 5.03%	2.42%*	0.39, 4.48%	− 3.35%*	− 6.21, − 0.41%	− 4.25%*	− 7.49, − 0.89%	1.47%	− 1.99, 5.05%
Specified Both Coaches and Parents/Guardian Education	1.65%*	0.87, 2.43%	2.59%*	0.26, 4.99%	0.99%	− 0.96, 2.97%	− 1.15%*	− 1.55, − 0.75%	− 2.10%	− 4.16, 0.01%	0.02%	− 0.36, 0.39%
Specified Either Coaches or Parents/Guardian Education	2.21%*	0.96, 3.47%	8.57%*	1.69, 15.92%	4.93%	− 0.22, 10.35%	− 1.04%*	− 1.71, − 0.38%	− 6.20%*	− 11.02, − 1.12%	− 0.54%	− 1.13, 0.05%

*Abbreviations*: *RTP* Return to play, *TBI* Traumatic brain injury, *CI* Confidence interval

* *P* < 0.05

^a^ Additional rate of change per standardized month

^b^ Difference in rate of change per standardized month relative to incident concussion rates

#### Return to play clearance requirements

**Fig. 2:**
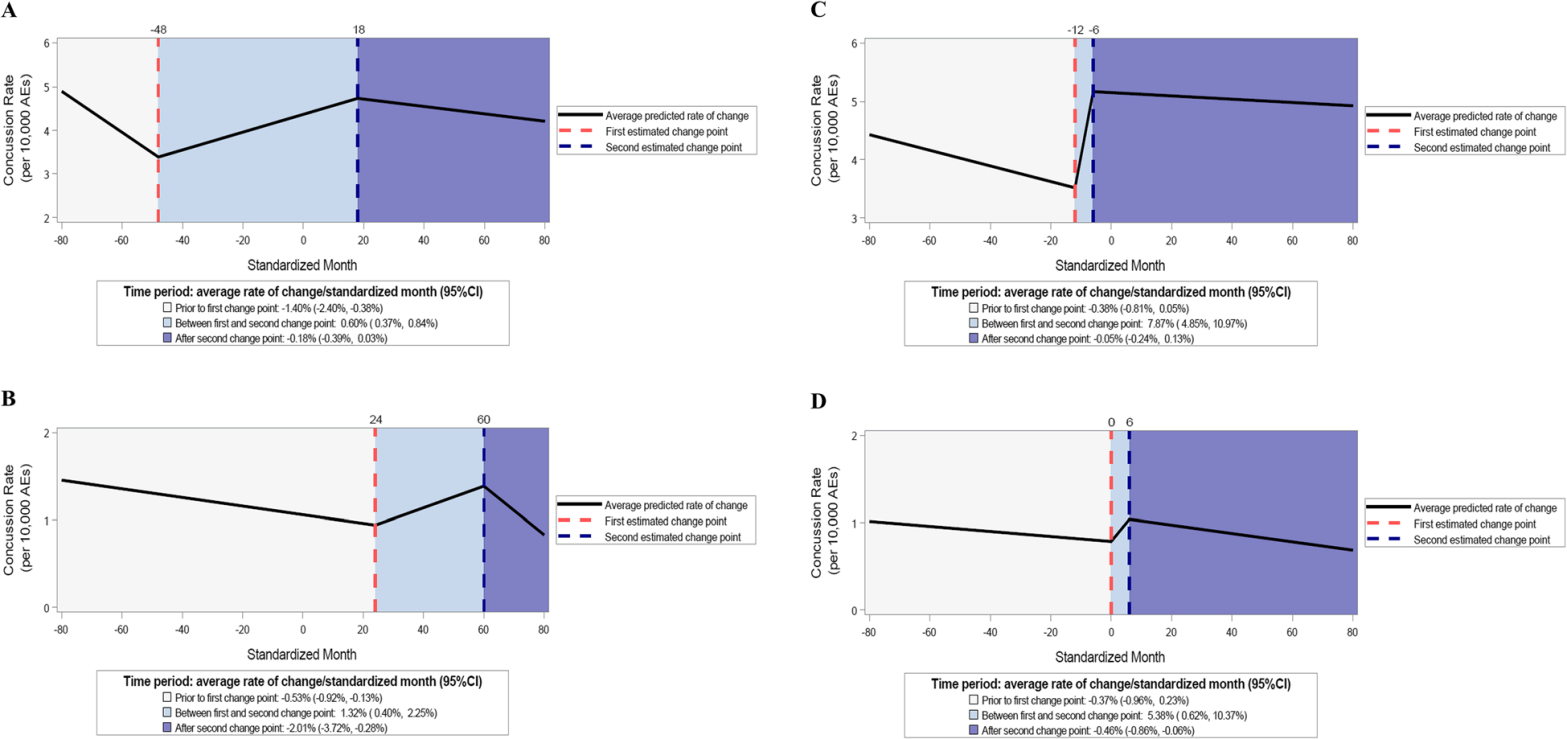
Estimated trends by TBI law language regarding healthcare provider RTP clearance requirements, 2005/06–2017/18. **a** Incident concussion rates-specified category of healthcare provider; **b** recurrent concussion rates-specified category of healthcare provider; **c** incident concussion rates-category of healthcare provider not specified; **d** recurrent concussion rates-category of healthcare provider not specified

Among states with TBI laws specifying the category of healthcare provider for RTP clearance, incident concussion rates significantly increased prior to law passage and decreased after law passage (Fig. 2a). Recurrent concussion rates were observed to significantly increase and decrease after law passage (Table 2 and Fig. 2b).

**Fig. 3:**
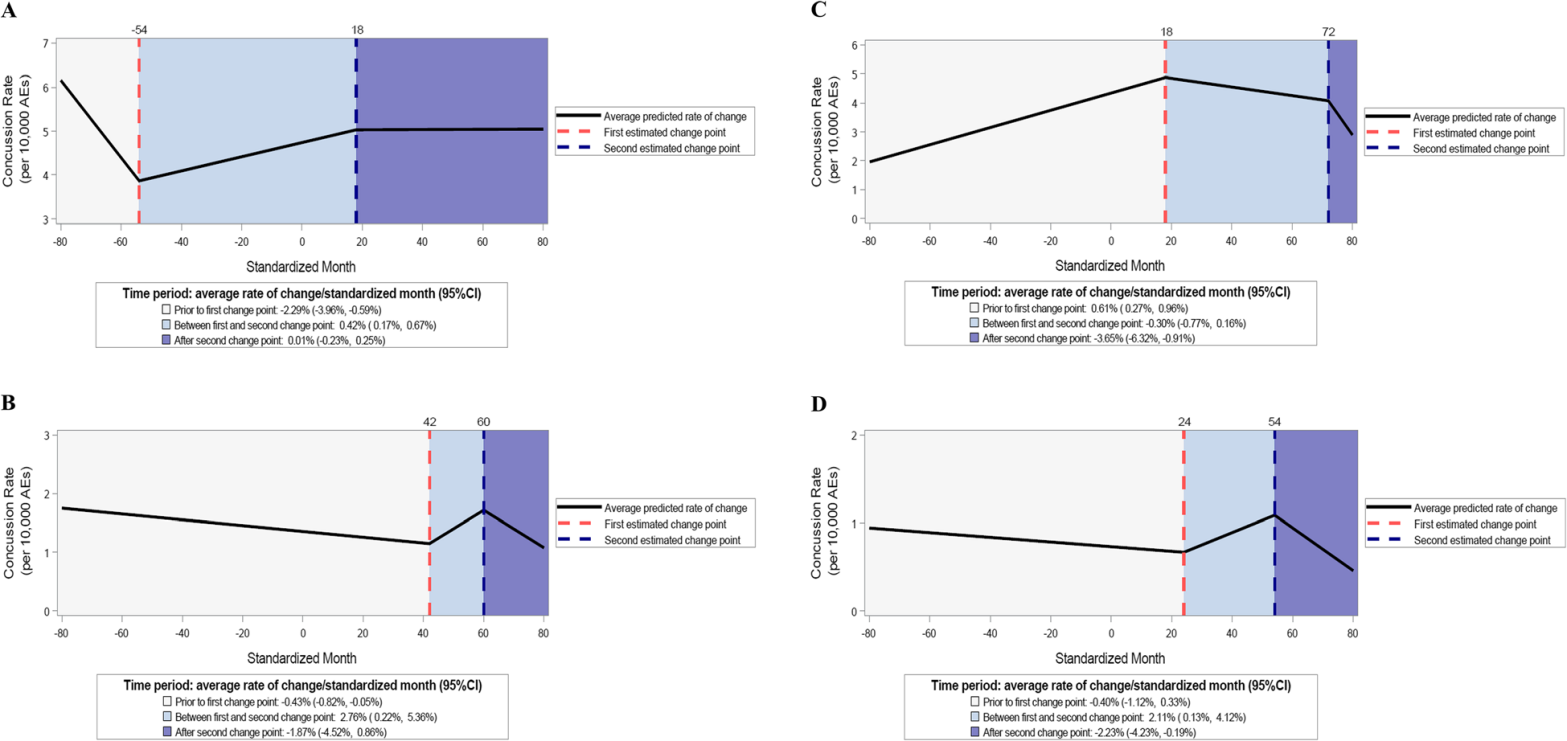
Estimated trends by TBI law language regarding category of healthcare provider RTP clearance requirements, 2005/06–2017/18. **a** Incident concussion rates-multiple categories of healthcare providers; **b** recurrent concussion rates-multiple categories of healthcare providers; **c** incident concussion rates-physicians only; **d** recurrent concussion rates-physicians only

Incident concussion rates significantly increased prior to law passage among states with TBI laws that allowed multiple categories of healthcare providers for RTP clearance. However, no significant changes in rates of incident concussions were observed during the post-law period (Table 2 and Fig. 3a). While recurrent concussion rates were observed to significantly increase after law passage, the decline in recurrent concussion rates during the post-law period was not statistically significant (Table 2 and Fig. 3b).

Among states with TBI laws that specified only physicians were eligible to provide RTP clearance, incident concussion rates significantly decreased on average by an additional 0.91%/STDM (95%CI: − 1.61, − 0.21%) and significantly decreased again by an additional 3.35%/STDM (95%CI: − 6.21, − 0.41%) during the post-law period (Table 2 and Fig. 3c).

#### Education requirements

**Fig. 4:**
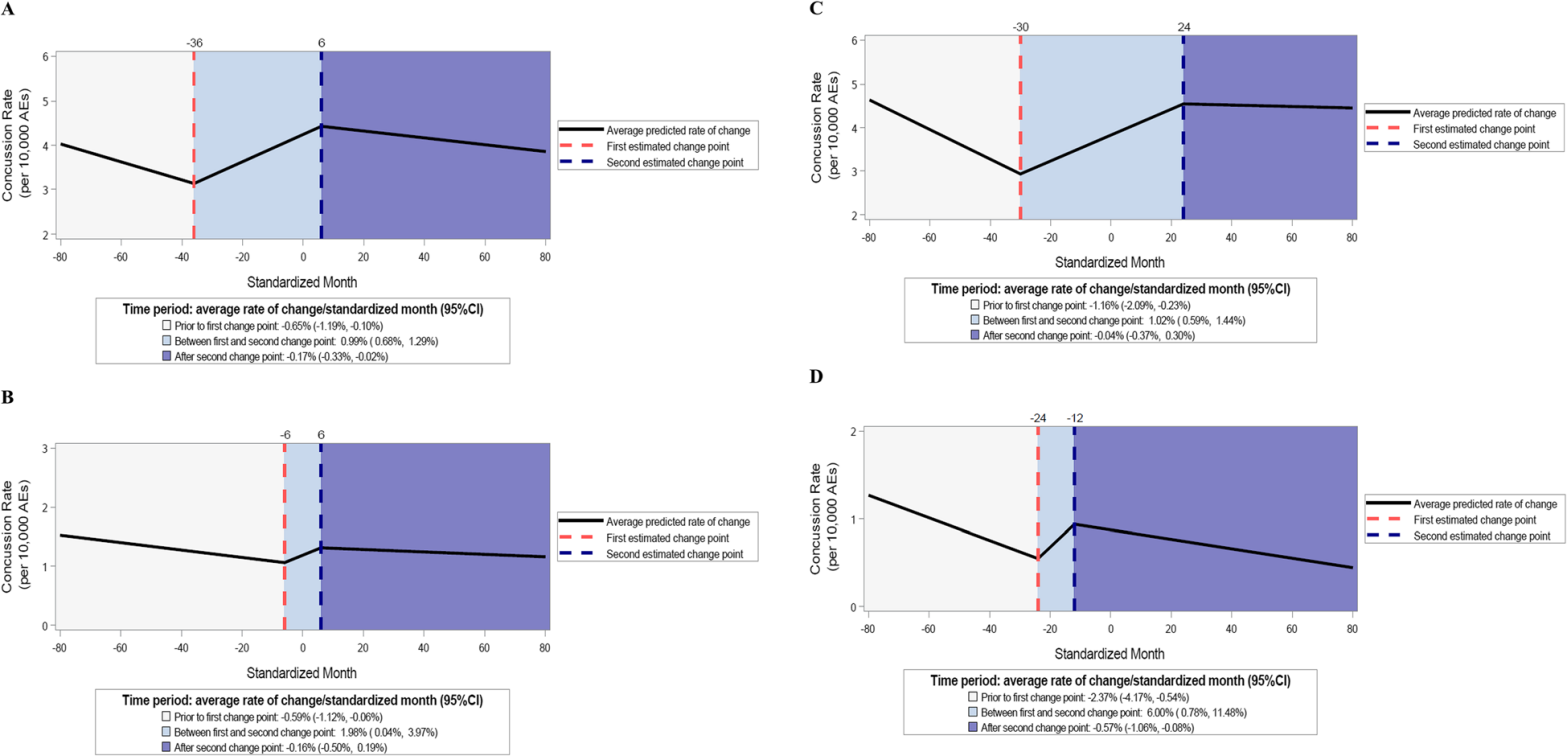
Estimated trends by TBI law language regarding requirements for education, 2005/06–2017/18. **a** Incident concussion rates-both coaches and parents/guardians; **b** recurrent concussion rates-both coaches and parents/guardians; **c** incident concussion rates-either coaches or parents/guardians; **d** recurrent concussion rates-either coaches or parents/guardians

For states with TBI laws that required education regarding concussion signs and symptoms for both coaches and parents/guardians, both incident and recurrent concussion rates were observed to significantly increase prior to law passage and decrease after law passage. Additionally, no significant differences were observed when comparing incident vs. recurrent concussion rates before or after law passage. Similar trends were observed among states with TBI laws that required education regarding concussion signs and symptoms for either coaches or parents/guardians (Table 2 and Fig. 4).

### Legislation language group comparisons

#### Return to play clearance requirements

Comparing states that specified vs. states that did not specify the category of healthcare provider for RTP clearance, no significant differences were observed for trends of recurrent concussion rates (Table 3). Although not statistically significant, the rate of decline during the post-law period for recurrent concussion rates was 1.59%/STDM (95%CI: − 3.42, 0.22%) greater among states that specified the category of healthcare provider compared to states that did not specify the category of healthcare provider for RTP clearance.

**Table 3:** Comparisons of Specific Language of TBI Laws by Time Period and Injury Characterization, 2005/06–2017/18

**Comparisons**	**After First Change Point**				**After Second Change Point**			
	**Incident**	**95%CI**	**Recurrent**	**95%CI**	**Incident**	**95%CI**	**Recurrent**	**95%CI**
Did Not Specify Category of Healthcare Provider for RTP Clearance vs. Specified Category of Healthcare Provider^a^	7.22%*	4.22, 10.31%	4.01%	− 0.77, 9.02%	0.12%	− 0.15, 0.40%	1.59%	− 0.22, 3.42%
Specified Multiple Categories of Healthcare Providers vs. Specified Physicians Only for RTP Clearance^b^	0.73%*	0.19, 1.26%	0.64%	− 2.49, 3.87%	3.79%*	0.92, 6.75%	0.37%	− 2.99, 3.86%
Specified Both Coaches and Parents/Guardians vs. Specified Either Coaches or Parents/Guardians Education^c^	− 0.03%	− 0.55, 0.49%	− 3.79%	− 8.82, 1.52%	− 0.14%	− 0.51, 0.23%	0.42%	− 0.18, 1.02%

*Abbreviations*: *RTP* Return to play, *TBI* Traumatic brain injury, *CI* Confidence interval

* *P* < 0.05

^a^ Difference in rate of change per standardized month relative to states that specified category of healthcare provider for return to play clearance

^b^ Difference in rate of change per standardized month relative to states that specified physician only for return to play clearance

^c^ Difference in rate of change per standardized month relative to states that specified either coaches or parents/guardians education

When comparing states that specified physicians only vs. states that allowed multiple categories of healthcare providers to provide RTP clearance, a significant difference was only observed when comparing trends in incident concussion rates (Table 3). Comparing the initial change in incident concussion rates, states with TBI laws that allowed multiple categories of healthcare providers increased on average by an additional 0.73%/STDM (95%CI: 0.19, 1.26%) compared to states with TBI laws that specified physicians only for RTP clearance (Table 3). Furthermore, comparing the subsequent change in incident concussion rates during the post-law period, states with TBI laws that specified physicians only decreased on average by an additional 3.79%/STDM (95%CI: − 6.75, − 0.92%) compared to states with TBI laws that allowed multiple categories of healthcare providers for RTP clearance. Although not statistically significant, the decline in recurrent concussion rates during the post-law period among states that specified physicians only for RTP clearance was 0.37%/STDM (95%CI: − 3.86, 2.99%) greater compared to states that allowed multiple categories of healthcare providers for RTP clearance.

#### Education requirements

Rates of incident and recurrent concussions over time stratified by who is required to complete education regarding concussion signs and symptoms demonstrated no significant differences when comparing states that specified coaches and parents/guardians vs. coaches or parents/guardians education (Table 3).

## Discussion

A comprehensive understanding of the nationwide effectiveness of state TBI laws is important given the growing population of high school athletes. This study was the first, to our knowledge, to evaluate trends in high school sports-related incident and recurrent concussion rates in relation to what category of healthcare provider is legally allowed to provide RTP clearance for athletes and who is required to complete education regarding concussion signs and symptoms.

Our findings partially support our a priori hypotheses. Overall incident and recurrent concussion rates significantly increased prior to law passage, and incident concussion rates plateaued during the post-law period. Although overall recurrent concussion rates were observed to decrease in the post-law period, the decrease was not statistically significant. These findings were consistent with the other two nationwide studies (Yang et al. 2017; Gibson et al. 2015). However, our results contradicted findings from two single state studies that reported no significant change in concussion rates after TBI law passage (O’Kane et al. 2014; LaRoche et al. 2016). Differences could be attributed to the small sample sizes, study population, and relatively short time period of evaluation observed with the prior single-state studies. The increase in concussion rates observed during the pre-law period could be attributed to grassroot efforts, educational campaigns, and national media coverage around sports-related concussions that occurred prior to law passage (Centers for Disease Control and Prevention 2019; Schwarz 2019). The observed increase in concussion rates prior to law passage is likely not an actual increase in the underlying incidence of concussions, but rather the effect of educational and media efforts during the pre-law period, resulting in increased knowledge, recognition, reporting, and diagnoses of concussions.

Our findings indicate that state TBI laws may be associated with a greater, albeit non-significant, post-law impact on recurrent compared to incident concussions. Studies have suggested that preventing injured athletes from returning to play until adequate recovery may reduce the risk of subsequent injuries (Committee on Sports-Related Concussions in Youth 2014; Halstead and Walter 2010; Boden et al. 2007). Intuitively, the RTP components of TBI laws should not affect the risk of incident concussions, as reflected in the near zero non-significant post-law rate of decline in overall incident concussion rates observed in this study. Results of our study suggest that the RTP components of the law are effective as a secondary rather than a primary concussion intervention. Continued primary intervention efforts, such as contact-practice limitations and rule changes should be applied alongside state TBI laws to attenuate the risk of both incident and recurrent concussions.

We hypothesize that healthcare providers specifically named in the respective TBI laws may be more inclined to review current gold standards of concussion diagnosis and RTP protocols compared to healthcare providers in states with ambiguity in their TBI laws regarding the category of healthcare provider. No significant differences were observed between post-law incident and recurrent concussion rates among states that did not specify the category of healthcare provider for RTP clearance. However, the post-law rate of decline was significantly greater for recurrent compared to incident concussion rates among states that specified the category of healthcare provider for RTP clearance. Furthermore, among states that specified the category of healthcare provider for RTP clearance, the post-law rate of decline in recurrent concussion rates was more than four times the rate compared to states that did not specify the category of healthcare provider for RTP clearance. Although this difference was not statistically significant, it is suggestive of a greater magnitude of decline in recurrent concussion rates among states with TBI laws that specified the category of healthcare provider to provide clearance for an injured athlete’s return to play. The results suggest that specifying the healthcare provider for RTP clearance may be associated with a greater impact on reducing the risk of recurrent concussions. These findings indicate specifying the category of healthcare provider eligible to provide RTP clearance may increase the effectiveness of state TBI laws.

Comparing trends of incident concussion rates between the specific categories of healthcare providers for RTP clearance showed a significantly greater initial increase in incident concussion rates among states that allowed multiple categories of healthcare providers for RTP clearance and a significantly greater decline during the post-law period among states that specified physicians only for RTP clearance. The flip in direction of associations after the change points suggest a potential underreporting of incident concussions among states with TBI laws that specified physicians only for RTP clearance. Access to physicians may play a role in concussion reporting behaviors. A study that assessed concussion reporting behaviors among high school athletes with and without access to an AT found that in schools without an AT, athletes were more than 3 times as likely to not report a concussion because they did not want to go to a doctor and almost 4 times as likely to not report a concussion due to not having health insurance (Wallace et al. 2017). The results suggest that restricting RTP clearance requirements to physicians only may result in increased unreported concussions, leaving injured athletes without care and placing athletes at increased risk of recurrent concussions.

Assumptions that physicians are the most qualified in concussion evaluation and management may be inaccurate; some non-physician healthcare professionals (e.g., school ATs, school nurses, physical therapist at concussion clinics, etc.) may be more knowledgeable and/or may possess more up-to-date concussion training than some general physicians who rarely treat concussed youth athletes (Itriyeva et al. 2017; Zonfrillo et al. 2012). The small magnitude and lack of statistical significance observed for post-law recurrent concussion rates, coupled with the potential underreporting of incident concussions that is likely attributed to access to healthcare and reporting behaviors suggests that restricting clearance requirements to physicians only may not be the most effective approach for TBI legislation. Our results suggest that states should allow multiple categories of healthcare providers to provide RTP clearance, instead of restricting the requirements to physicians only.

### Strengths and limitations

Strengths of this study include the use of the High School RIO database, which allowed for a nationwide assessment of longitudinal trends in high school sports-related concussion rates in relation to state TBI law passage. Stratification by state level variations in legislation’s language was another strength, as it was not assessed in previous studies. The ability to distinguish between incident and recurrent concussions was vital in evaluating concussion trends, as TBI laws function as a secondary rather than a primary concussion intervention. Finally, the profile likelihood method allowed for the identification of change points without need of a priori knowledge or assumptions regarding the timing of the shift in the piecewise regression models.

This study has several limitations. Schools participating in High School RIO may not be representative of all high schools as only schools with an AT were eligible. Concussion rates may be higher in High School RIO schools as an AT’s presence potentially increases the likelihood of concussion recognition. While an AT’s participation may limit generalizability, it improves data quality. Because the profile likelihood method identifies the optimal change points in the dataset, there may be an issue of overestimated precision of the parameter estimates leading to type I error inflation. A non-parametric bootstrapping approach was attempted to derive unbiased standard errors. However, due to highly influential observations, the bootstrap distributions for all 14 models of interest were found to be asymmetrical and heavily skewed. This resulted in unusable bootstrap standard errors and percentile confidence intervals. Therefore, the results presented in this study represent unadjusted standard errors that are smaller and confidence intervals narrower than they likely should be. Future studies should address the overestimated precision issues by potentially utilizing a parametric bootstrapping approach. Finally, we did not account for any state-specific amendments made to the law since initial TBI law passage, which may have affected post-law concussion rates. Future studies should account for the specific language of TBI laws as a potential time-varying covariate. Additionally, future studies might consider changes between date of law passage and date of implementation of TBI laws as well as potential waning compliance with legislation years after implementation. Despite these limitations, this study was the first to evaluate associations between the specific language used in state TBI laws and observed trends in incident and recurrent concussion rates.

## Conclusions

We found an increase in overall concussion rates prior to law passage and a decrease in rates during the post-law period for both incident and recurrent concussions. Trends in incident and recurrent concussion rates were observed to differ by state level variations in legislation’s language, specifically for language relating to category of healthcare provider for RTP clearance. States may benefit from specifying the category of healthcare provider for RTP clearance in their respective TBI laws, if they have not done so already. Additionally, specifying physicians only for RTP clearance, although intuitively reasonable, may not be necessary and may result in underreporting of concussions. States may benefit from allowing multiple categories of healthcare providers to provide RTP clearance. The findings may have implications for state lawmakers considering revisions to current TBI legislation.

## Data Availability

The dataset analyzed in the current study is available by request from Dr. R. Dawn Comstock, Director of the National High School Sports Related Injury Surveillance Study (High School RIO) from 2005/06–2018/19. Additional information regarding High School RIO, including annual summary reports, is available at http://www.ucdenver.edu/academics/colleges/PublicHealth/research/ResearchProjects/piper/projects/RIO/Pages/Study-Reports.aspx

## Abbreviations

US: United States
TBI: Traumatic Brain Injury
RTP: Return to Play
AT: Athletic Trainer
High School RIO: National High School Sports-Related Injury Surveillance Study
AE: Athletic Exposure
STDM: Standardized Month
CI: Confidence Interval
